# Effects of *FADS* and *ELOVL* polymorphisms on indexes of desaturase and elongase activities: results from a pre-post fish oil supplementation

**DOI:** 10.1007/s12263-014-0437-z

**Published:** 2014-11-04

**Authors:** Hubert Cormier, Iwona Rudkowska, Simone Lemieux, Patrick Couture, Pierre Julien, Marie-Claude Vohl

**Affiliations:** 1 Institute of Nutrition and Functional Foods (INAF), Laval University, 2440 Hochelaga Blvd, Québec, QC G1V 0A6 Canada; 2 School of Nutrition, Laval University, Quebec City, G1K 7P4 Canada; 3 Department of Kinesiology, Laval University, Quebec City, G1V 0A6 Canada; 4 Endocrinology and Nephrology, CHU de Québec Research Center, Québec, G1V 4G2 Canada

**Keywords:** Omega-3 fatty acids, Diet and dietary lipids, Genomics, Fish oil, Omega-6 fatty acids, Genotype

## Abstract

Polymorphisms (SNPs) within the *FADS* gene cluster and the *ELOVL* gene family are believed to influence enzyme activities after an omega-3 (n-3) fatty acid (FA) supplementation. The objectives of the study are to test whether an n-3 supplementation is associated with indexes of desaturase and elongase activities in addition to verify whether SNPs in the *FADS* gene cluster and the *ELOVL* gene family modulate enzyme activities of desaturases and elongases. A total 208 subjects completed a 6-week supplementation period with 5 g/day of fish oil (1.9–2.2 g/day of EPA + 1.1 g/day of DHA). FA profiles of plasma phospholipids were obtained by gas chromatography (*n* = 210). Desaturase and elongase indexes were estimated using product-to-precursor ratios. Twenty-eight SNPs from *FADS1*, *FADS2*, *FADS3, ELOVL2* and *ELOVL5* were genotyped using TaqMan technology. Desaturase indexes were significantly different after the 6-week n-3 supplementation. The index of δ-5 desaturase activity increased by 25.7 ± 28.8 % (*p* < 0.0001), whereas the index of δ-6 desaturase activity decreased by 17.7 ± 18.2 % (*p* < 0.0001) post-supplementation. Index of elongase activity decreased by 39.5 ± 27.9 % (*p* < 0.0001). Some gene–diet interactions potentially modulating the enzyme activities of desaturases and elongases involved in the FA metabolism post-supplementation were found. SNPs within the *FADS* gene cluster and the *ELOVL* gene family may play an important role in the enzyme activity of desaturases and elongases, suggesting that an n-3 FAs supplementation may affect PUFA metabolism.

## Introduction

**Fig. 1:**
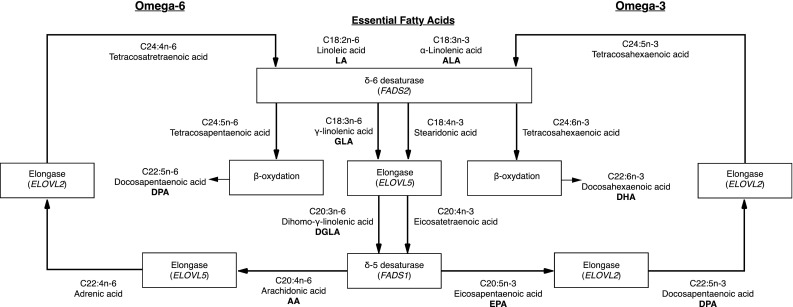
Synthesis of n-3 and n-6 PUFAs in humans. Elongases and desaturases are the enzymes responsible for the conversion of α-linolenic acid (ALA, C18:3n-3) to eicosapentaenoic acid (EPA, C20:5n-3) and docosahexaenoic acid (DHA, C22:6n-3). They are also involved in the synthesis of omega-6′s products, mainly the arachidonic acid (AA, C20:4n-6) which leads to the production of proinflammatory mediators. Desaturases and elongases are both involved in the fatty acid metabolism. These enzymes are encoded by genes from the *FADS* gene cluster and the *ELOVL* gene family

Elongation and desaturation of long-chain (LC) polyunsaturated fatty acids (PUFA) of the omega-3 (n-3) family are made possible by enzymes called desaturases and elongases. Figure 1 shows the metabolic pathway of n-3 and omega-6 (n-6) fatty acids (FA) with an emphasis on enzymes required for synthesis of LC-PUFAs. The use of the δ-6 desaturases (D6D) twice in the conversion of alpha-linolenic acid (ALA) to docosahexaenoic acid (DHA) suggests that this enzyme may play a key regulatory role in the PUFA metabolism (Portolesi et al. 2007).

Fatty acid desaturase 1 and 2 (*FADS1* and *FADS2*, respectively) genes encode for key enzymes in the PUFA metabolism, the δ-5 desaturase (D5D) and D6D, respectively (Malerba et al. 2008). These desaturases are responsible for the double bond formation between two carbons leading to more unsaturated FAs. Elongases are encoded by genes within the *ELOVL* family and catalyzes the elongation of the aliphatic chain of carbons leading to the formation of LC-PUFAs (Jakobsson et al. 2006). Two elongases are involved in LC-PUFA synthesis, the fatty acid elongase-2 and the fatty acid elongase-5, encoded, respectively, by *ELOVL2* and *ELOVL5* genes. A high desaturase activity may lead to an increased bioavailability of arachidonic acid (AA) with dominant synthesis of AA-derived proinflammatory eicosanoids, possibly leading to vascular damage, especially in populations eating a Western diet (Martinelli et al. 2009). Excessive amounts of n-6 FAs from the diet and from the endogenous synthesis, as seen in today’s Western diets, promote the pathogenesis of many diseases including cardiovascular disease, cancers as well as inflammatory and autoimmune diseases, whereas increased levels of n-3 FA (a lower n-6/n-3 ratio) exert suppressive effects (Simopoulos 2008). In opposition to a diet rich in n-6 PUFA, a diet rich in n-3 FAs could result in a preferential synthesis of anti-inflammatory eicosanoids in addition to a high desaturase activity (Martinelli et al. 2009). A shift from n-6 to n-3 induces changes in the eicosanoid profile, which may lead to a decrease inflammatory state (Calder 2009).

There is a high affinity of D6D for ALA (D’Andrea et al. 2002), thus favouring the n-3 FA pathway at the expense of the n-6 pathway (Sprecher 2002). These two classes of FAs compete for a number of enzyme systems which can influence inflammatory responses, vascular reactivity, and platelet aggregation (Harris 2006). Moreover, oleic acid (18:1n-9) from the omega-9 (n-9) pathway is also metabolized by D5D and D6D (Das 2007). As a result, all these series of FAs, i.e., n-3, n-6, and n-9, compete for the same set of enzymes, although a preferential affinity has been demonstrated according to the following sequence: n-3 > n-6 > n-9 (Das 2007).

Recent studies suggested that some common SNPs of the *FADS* gene cluster and of the *ELOVL* gene family (Tanaka et al. 2009) are associated with plasma n-6 and n-3 FA levels. Accordingly, Lemaitre et al. (2011) have shown, in a genome-wide association studies in five population-based cohorts comprising 8,866 subjects of European ancestry, that SNPs from the *FADS* gene cluster and from *ELOVL2* gene were associated with plasma eicosapentaenoic acid, DHA and ALA levels measured in plasma phospholipids. SNPs within the *FADS* gene cluster, in particular rs175547, are also believed to alter desaturase activity in two different ethnic populations, Caucasian and Asian, as shown by Merino et al. (2011).

The objectives of the present study are to test whether n-3 FA supplementation is associated with indexes of desaturase and elongase activities as determined by product-to-precursor ratios in addition to verify whether SNPs in the *FADS* gene cluster and the *ELOVL* gene family modulate enzyme activities of desaturases and elongases following an n-3 FA supplementation.

## Materials and methods

### Sociodemographics

Two hundred and fifty-four unrelated subjects from the greater Quebec City metropolitan area were recruited between September 2009 and December 2011 through advertisements in local newspapers as well as by electronic messages sent to university students/employees. To be eligible for the study, subjects had to be aged between 18 and 50 years with a body mass index (BMI) between 25 and 40 kg/m^2^. They had to be non-smokers and free of any thyroid or metabolic disorders such as hypo/hyperthyroidism, dyslipidemia, and type 2 diabetes among others. Subjects were excluded if they had taken fish oil supplements for at least 6 months prior to the beginning of the study and if their baseline triglyceride levels were above 4.0 mmol/l. Statistical analyses were performed on 210 individuals who completed the supplementation period.

### Study design

The complete study design has been previously reported (Cormier et al. 2012). Briefly, subjects followed a run-in period of 2 weeks where a trained registered dietitian gave individual dietary instructions. Recommendations were drawn from the *Canada’s Food Guide to Healthy Eating* (Eating Well with Canada’s Food Guide 2007). After a 2-week run-in period, each participant received a bottle containing n-3 FA capsules (Ocean Nutrition, Nova Scotia, Canada) covering the following 6-week period. They had to take five capsules per day, which gave them a total of 3–3.3 g of n-3 FA (1.9–2.2 g EPA and 1.1 g DHA) per day. Subjects had to report any deviations that may have occurred during the protocol. They also had to write down their alcohol and fish consumption on a log sheet. Before each phase of the study, subjects received written and oral dietary instructions by a registered dietitian.

### SNPs selection and genotyping

SNPs in *FADS1*, *FADS2*, *FADS3, ELOVL2* and *ELOVL5* were identified using the International HapMap Project SNP database, based on the NCBI B36 assembly Data Rel 28. phase II + III, build 126. The *FADS* gene cluster is made of three genes that are located very close to each other among chromosome 11. Because of the head-to-head orientation of *FADS1* and *FADS2* and the tail-to-tail orientation of *FADS2* and *FADS3*, we added 500-kilo base pairs (kbp) downstream of *FADS1* and 2,500-kbp upstream of *FADS3* to cover the 5′UTR and 3′UTR regions. Intergenic areas comprised between *FADS1* and *FADS2* and between *FADS2* and *FADS3* were also covered. *ELOVL2* and *ELOVL5* genes are both located on chromosome 6. Gene Tagger procedure in Haploview V4.2 was used to determine tag SNPs using a minor allele frequency ≥1 % and pairwise tagging (*r* ^2^ ≥ 0.8). Subsequently, we examined linkage disequilibrium out of the nineteen SNPs of the *FADS* gene cluster area, the 4 tSNPs of the *ELOVL5* gene, and the 6 tSNPs of the *ELOVL2* gene using the LD Plot procedure in Haploview V4.2. The SIGMA GenElute Gel Extraction Kit (Sigma-Aldrich Co. St.Louis. Missouri. USA) has been used to extract genomic DNA. Selected SNPs of the *FADS* gene cluster (rs174456, rs174627, rs482548, rs2072114, rs12807005, rs174448, rs2845573, rs7394871, rs7942717, rs74823126, rs174602, rs498793, rs7935946, rs174546, rs174570, rs174579, rs174611, rs174616 and rs968567) and of the *ELOVL* genes (rs209492, rs2073040, rs2294852, rs9370194, rs13204015, rs12195587, rs4532436, rs3734397, rs2281591 and rs3798710) have been genotyped using validated primers and TaqMan probes (Life Technologies Corporation, Burlington, On.) (Livak 1999). DNA was mixed with TaqMan Universal PCR Master Mix (Life Technologies Corporation, Burlington, On.), with a gene-specific primer and with probe mixture (predeveloped TaqMan SNP Genotyping Assays; Life Technologies Corporation, Burlington, On.) in a final volume of 10 μl. Genotypes were determined using a 7500 RT-PCR System and analyzed using ABI Prism SDS version 2.0.5 (Life Technologies Corporation, Burlington, On.).

### Measurement of FA composition in plasma phospholipids

Subjects arrived at the research center following an overnight fast. FA composition of plasma phospholipids was determined by gas chromatography. Venous blood was drawn into EDTA tubes, and plasma was immediately separated by centrifugation at 500 g for 6 min and stored at −80 °C for subsequent analyses. Plasma lipids were extracted with chloroform:methanol (2:1, by volume) according to a modified Folch method (Shaikh and Downar 1981). Total phospholipids were then isolated with isopropyl ether:acetic acid (96:4) by thin layer chromatography (Lepage and Roy 1986). Isolated plasma phospholipids were then methylated (Lepage and Roy 1986). FA profiles were obtained after methylation in methanol/benzene 4:1 (v/v) (Lepage and Roy 1986) and capillary gas chromatography using a temperature gradient on a HP5890 gas chromatograph (Hewlett Packard, Toronto, Canada) equipped with a HP-88 capillary column (100 m × 0.25 mm i.d. × 0.20 µm film thickness; Agilent Technologies, Palo Atto, CA) coupled with a flame ionization detector (FID). Helium was used as carrier gas (split ratio 1:80). FA were identified according to their retention time, using the following standard mixtures as a basis for comparison: the FAME 37 mix (Supelco Inc., Bellefonte, PA) and the GLC-411 FA mix (NuChek Prep Inc, Elysian, MN), as well as the following methylated FAs C22:5n-6 (Larodan AB, Malmö, Sweden) and C22:5n-3 (Supelco Inc., Bellefonte, PA). Phospholipids FA profiles were expressed as the relative percentage areas of total FAs. The database and ontology of Chemical Entities of Biological Interest (ChEBI) has been used to identify FAs herein.

Estimates of D5D and D6D activities were computed using product-to-precursor ratios: (C20:4n-6, AA):(C20:3n-6, dihomo-γ-linolenic acid (DGLA)), and (C20:3n-6, DGLA):(C18:2n-6, linoleic acid (LA)), respectively, as previously described (Bokor et al. 2010). The index of elongase activity was calculated using the (C22:4n-6):(C20:4n-6, AA) ratio.

### Statistical analysis

All genotype distributions were tested for any deviation from Hardy–Weinberg equilibrium (HWE) using the ALLELE procedure in S.A.S Genetics v9.3 (S.A.S Institute Inc., Cary, North Carolina, USA). Significance testing for linkage disequilibrium coefficient D was obtained using a Chi-square test, likelihood ratio and Fisher’s exact test (*p* ≤ 0.01). All other statistical analyses were carried out using S.A.S statistical software v9.3 (S.A.S Institute Inc., Cary, North Carolina, USA). Normal distribution was evaluated using the box-plot, as well as skewness and kurtosis ranges. When needed, variables non-normally distributed were log_10_-transformed. A linear regression using the stepwise bidirectional elimination approach was used to assess which SNPs could explain part of the enzyme activity’s variance where the effects of the SNPs, age, sex, and BMI were included in the statistical model and where the effect of baseline enzyme activity was added (in post-supplementation only). The MIXED procedure was used to test for the effects of the genotype, the supplementation (effect of time) and the genotype × supplementation interaction for each SNP on estimates of enzyme activities when age, sex and BMI were included in the model. The repeated statement was used to indicate the within subjects (repeated) variables. Genotype groups were assessed as three groups expressed as major allele homozygotes, heterozygotes and minor allele homozygotes. For some SNPs, heterozygotes and minor allele homozygotes were grouped if the genotype frequency was under 5 %. The statistical significance was defined as *p* ≤ 0.05. Since polymorphisms tested in complex diseases rarely account for a large proportion of the variance, results are thus presented before correction for multiple testing and using a *p* ≤ 0.05.

## Results

Subjects’ characteristics pre- and post-supplementation are presented in Table 1. Estimates of D5D activity increased by 25.7 ± 28.8 % (Mean ± SD, *p* < 0.0001), whereas surrogate estimates of D6D activity decreased by 17.7 ± 18.2 % (*p* < 0.0001) after the supplementation. Index of elongase activity decreased by 39.5 ± 27.9 % (*p* < 0.0001) (Table 1). Triglyceride, insulin, total-cholesterol and LDL-cholesterol levels were inversely associated with D5D activity and positively associated with D6D activity after the supplementation (data not shown, *p* < 0.008 for all). Moreover, a decrease in plasma levels of n-6 FAs was observed: LA (−9.85 ± 10.76 %, *p* < 0.0001); AA (−11.20 ± 9.99 %, *p* < 0.0001); as well as a decrease in plasma levels of ALA (−31.44 ± 50.44 %, *p* = 0.0002) following the intervention. On the n-3 FAs side, an increase was observed for plasma levels of EPA (+331.11 ± 214.74 %, *p* < 0.0001) and DHA (+46.31 ± 27.46 %, *p* < 0.0001). The same pattern was also observed for absolute quantities of FAs (μg/ml) where similar rates of change were revealed as shown in Table 1.

**Table 1:** Characteristics of the subjects

	Pre-n-3 PUFA supplementation	Post-n-3 PUFA supplementation	Δ	*p* value
Sex (men/women)	97/113			
Age (years)	30.8 ± 8.7			
Weight (kg)	81.3 ± 13.9	81.6 ± 14.2		0.82
BMI (kg/m^2^)	27.8 ± 3.7	27.9 ± 3.8		0.81
Plasma desaturase activity				
D5D (20:4n-6/20:3n-6)	3.57 ± 1.15	4.34 ± 1.25	25.71 ± 28.83 (*n* = 210)	<0.0001
D6D (20:3n-6/18:2n-6)	0.17 ± 0.05	0.14 ± 0.04	−17.68 ± 18.15 (*n* = 210)	<0.0001
Plasma elongase activity				
ELOVL2 (22:4n-6/20:4n-6)	0.0318 ± 0.0064	0.0193 ± 0.0093	−39.52 ± 27.89 (*n* = 209)	<0.0001
Omega-6 (% of total FAs)				
Linoleic acid, C18:2n-6, LA	19.71 ± 2.08	17.69 ± 2.22	−9.85 ± 10.76	<0.0001
Arachidonic acid, C20:4n-6, AA	11.05 ± 1.74	9.74 ± 1.44	−11.20 ± 9.99	<0.0001
Omega-3 (% of total FAs)				
Alpha-linolenic acid, C18:3n-3, ALA	0.18 ± 0.15	0.14 ± 0.12	−31.44 ± 50.44	0.0002
Eicosapentaenoic acid, C20:5n-3, EPA	1.12 ± 0.52	4.10 ± 1.28	331.11 ± 214.74	<0.0001
Docosahexaenoic acid, C22:6n-3, DHA	3.53 ± 0.77	5.03 ± 0.86	46.31 ± 27.46	<0.0001
Omega-6 (μg/ml)				
Linoleic acid C18:2n-6, LA	227.26 ± 44.46	199.16 ± 45.38	−11.63 ± 15.82	<0.0001
Arachidonic acid, C20:4n-6, AA	128.14 ± 32.77	109.63 ± 27.03	−13.04 ± 14.26	<0.0001
Omega-3 (μg/ml)				
Alpha-linolenic acid, C18:3n-3, ALA	2.22 ± 1.89	1.72 ± 1.52	−32.08 ± 51.42	<0.0001
Eicosapentaenoic acid, C20:5n-3, EPA	12.92 ± 6.41	46.15 ± 16.99	328.79 ± 227.51	<0.0001
Docosahexaenoic acid, C22:6n-3, DHA	40.97 ± 12.54	57.04 ± 16.05	43.55 ± 33.50	<0.0001

Measurements made prior to- and after a 6-week n-3 FA supplementation; values are mean ± SD

The rate of change (Δ) expressed as the relative variation of plasma FAs as a percentage (%) or absolute quantities (μg/ml) between the pre- and post-n-3 PUFA period. Differences between the pre- and post-n-3 PUFA supplementation were tested using a pairwise Student’s *t* test

All SNPs were in HWE except rs7935946 (*FADS*) that was not considered for further analyses. All selected SNPs are presented in Table 2 and LD tables are presented in Tables 3, 4, and 5.  To validate the presence of associations between surrogate estimates of enzyme activities involved in the n-3 FA synthesis pathway with SNPs of the *FADS* gene cluster or *ELOVL* gene family, all SNPs were included in a stepwise regression model adjusted for the effects of age, sex and BMI. Prior to the n-3 FA supplementation, three SNPs were associated with the estimates of D5D activity: rs968567 (*R* ^2^ = 13.83 %, *p* < 0.0001), rs2845573 (*R* ^2^ = 11.65 %, *p* < 0.0001) and rs7394871 (*R* ^2^ = 4.25 %, *p* < 0.0001), one SNP was associated with estimates of D6D activity: rs968567 (*R* ^2^ = 2.86 %, *p* = 0.01) and two SNPs were associated with the index of elongase activity: rs2281591 (*R* ^2^ = 3.21 %, *p* = 0.008) and rs3798710 (*R* ^2^ = 2.79 %, *p* = 0.01). To account for the 6-week supplementation, the same statistical model was used with data post-n-3 FA supplementation as the dependent variable, adjusted for baseline data, age, sex and BMI. With the use of the stepwise bidirectional selection method, five SNPs were associated with the post-supplementation estimates of D5D activity: rs174546 (*R* ^2^ = 8.26 %, *p* < 0.0001), rs968567 (*R* ^2^ = 2.47 %, *p* = 0.004), rs7935946 (*R* ^2^ = 2.42 %, *p* = 0.004), rs2072114 (*R* ^2^ = 2.92 %, *p* = 0.001) and rs12807005 (*R* ^2^ = 1.20 %, *p* = 0.04); one SNP was associated with post-supplementation estimates of D6D activity: rs12807005 (*R* ^2^ = 0.92 %, *p* = 0.04); and none of the SNPs were associated with the index of elongase activity after an n-3 FA supplementation (data not shown) (Tables 6, 7, 8).

**Table 2:** Characteristics of SNPs within the *FADS* gene cluster and *ELOVL* gene family

dbSNP no.^a^	Sequence^b^	Position	Alleles (major/minor)	*n* ^c^ (%)									MAF
				AA	CC	CA	CG	CT	GA	GG	GT	TT	
*FADS1*													
rs174546	TGC[**C/T**]TTG	3′UTR	C/T		103 (49.8)			86 (41.2)				10 (9.1)	*T* = 29.8
*FADS2*													
rs482548	CAC[**C/T**]GTG	3′UTR	C/T		161 (77.8)			40 (19.3)				6 (2.9)	*T* = 12.4
rs2072114	TTC[**A/G**]GGT	Intron	A/G	167 (79.9)					38 (18.2)	4 (1.9)			*G* = 10.9
rs2845573	TCA[**C/T**]GTT	Intron	A/G	177 (84.7)					30 (14.4)	2 (1.0)			*G* = 8.1
rs174602	CCC[**A/G**]TCC	Intron	T/C		9 (4.3)			59 (28.2)				141 (67.5)	*C* = 18.8
rs498793	AAC[**A/G**]CAG	Intron	C/T		62 (9.8)			99 (71.7)				43 (18.6)	*T* = 42.0
rs174570	TGA[**C/T**]GTA	Intron	C/T		159 (76.4)			46 (22.1)				3 (1.4)	*T* = 12.4
rs174579	TTT[**C/T**]CAG	Intron	C/T		127 (61.1)			78 (37.5)				3 (1.4)	*T* = 20.3
rs174611	GGA[**C/T**]CCT	Intron	T/C		12 (5.7)			84 (40.2)				113 (54.1)	*C* = 25.9
rs174616	TCA[**C/T**]GTT	Intron	G/A	51 (24.4)					158 (51.7)	50 (23.9)			*G* = 49.5
rs968567	CGG[**A/G**]AGC	5′UTR	G/A	2 (1.0)					63 (30.1)	144 (68.9)			*A* = 7.1
rs7935946	TTC[C/T]GGG	Intron			195 (93.3)			11 (5.3)				3 (1.4)	
*FADS3*													
rs174456	TAC[**A/C**]TGG	Intron	A/C	102 (48.8)	18 (8.6)	89 (42.6)							*C* = 30.0
rs7394871	GAC[**A/C**]CCT	Intron	C/A	2 (1.0)	181 (86.6)	26 (12.4)							*A* = 7.1
rs7942717	ACG[**A/G**]GTG	Intron	A/G	161 (77.0)					47 (22.5)	1 (0.5)			*G* = 11.7
Intergenic regions within the FADS gene cluster													
rs174627	CTG[**C/T**]GTA	Intergenic	G/A	2 (1.0)					48 (23.0)	159 (76.1)			*A* = 12.6
rs12807005	ATG[**A/G**]ATC	Intergenic	G/A	0 (0)					5 (2.4)	204 (97.6)			*A* = 1.2
rs174448	TGA[**C/T**]TTC	Intergenic	A/G	78 (37.5)					109 (52.4)	21 (10.1)			*G* = 36.3
rs7482316	CAA[**A/G**]CTG	Intergenic	A/G	168 (80.4)					39 (18.7)	2 (1.0)			*G* = 10.2
*ELOVL2*													
rs13204015	TTC[**C/T**]TTT	Intron	T/C		0 (0)			17 (8.1)				191 (91.9)	*C* = 4.1
rs12195587	AAC[**A/G**]TAA	Exon	G/A	1 (0.5)					59 (28.2)	149 (71.3)			*A* = 14.6
rs4532436	AGC[**C/G**]AAT	3′UTR	C/G		43 (21.0)		121 (59.0)			41 (20.0)			*G* = 49.5
rs3734397	CTC[**A/G**]GTA	3′UTR	C/G		129 (61.4)				69 (32.9)	15 (5.7)			*G* = 22.1
rs2281591	TCT[**A/G**]TTT	Intron	A/G	102 (48.6)					93 (44.4)	15 (7.1)			*G* = 29.3
rs3798710	TTT[**C/G**]AAC	Intron	C/G		139 (66.2)		62 (29.5)			9 (4.3)			*G* = 19.1
*ELOVL5*													
rs209492	TTG[**C/T**]TTA	Intron	T/C		4 (1.9)			47 (22.4)				159 (75.7)	*C* = 13.1
rs2073040	AGC[**A/G**]GAT	Intron	A/G	82 (39.1)					102 (48.6)	26 (12.4)			*G* = 36.7
rs2294852	CCA[**C/G**]GTT	Intron	C/G		59 (28.1)		103 (49.1)			48 (22.9)			*G* = 47.4
rs9370194	GAC[**C/T**]GTT	Intron	C/T		104 (49.5)			96 (45.7)				10 (4.8)	*T* = 27.6

*MAF* Minor allele frequency from the FAS cohort, calculated with the ALLELE procedure in SAS Genetics v9.3

^a^dbSNP no. from HapMap Data Rel 28 Phase II + III, August 10 on NCBI b36 Assembly dbSNP b126 database

^b^Genes sequences from dbSNP short genetics variations NCBI reference assembly

^c^Number of subjects for each genotype

**Table 3:** Linkage disequilibrium (*R* ^2^) of the tagging SNPs within the *FADS* gene cluster

	rs174546	rs12807005	rs968567	rs174570	rs2845573	rs2072114	rs174579	rs7935946	rs174602	rs498793	rs174611	rs174616	rs482548	rs174627	rs174448	rs7482316	rs7942717	rs7394871	rs174456
rs174546		0.016	0.424	0.304	0.159	0.223	0.568	0.054	0.229	0.04	0.452	0.287	0.047	0.232	0.363	0.067	0.021	0.037	0.222
rs12807005	0.016		0.007	0.005	0.003	0.004	0.01	0.001	0.007	0.02	0.003	0.001	0.029	0	0.004	0.003	0.002	0.002	0.001
rs968567	0.424	0.007		0.038	0.02	0.014	0.636	0	0.02	0.038	0.351	0.204	0.02	0.486	0.261	0.009	0.002	0.012	0.292
rs174570	0.304	0.005	0.038		0.522	0.345	0.028	0.005	0.1	0.01	0.033	0.035	0.014	0.013	0.037	0.124	0.008	0.177	0.001
rs2845573	0.159	0.003	0.02	0.522		0.714	0.029	0.003	0.021	0.03	0.026	0.016	0	0.008	0.015	0.154	0.007	0.357	0.002
rs2072114	0.223	0.004	0.014	0.345	0.714		0.04	0.242	0.004	0.001	0.036	0.031	0.008	0.006	0.037	0.191	0.018	0.249	0
rs174579	0.568	0.01	0.636	0.028	0.029	0.04		0.01	0.203	0.025	0.34	0.227	0.029	0.371	0.312	0.001	0.004	0.018	0.292
rs7935946	0.054	0.001	0	0.005	0.003	0.242	0.01		0.066	0.019	0.008	0.014	0.003	0	0.022	0.031	0.011	0.002	0.002
rs174602	0.229	0.007	0.02	0.1	0.021	0.004	0.203	0.066		0.002	0.11	0.123	0.021	0.005	0.199	0.094	0.085	0.013	0.034
rs498793	0.04	0.02	0.038	0.01	0.03	0.001	0.025	0.019	0.002		0.109	0.121	0.033	0.033	0.049	0.025	0.002	0.017	0.022
rs174611	0.452	0.003	0.351	0.033	0.026	0.036	0.34	0.008	0.11	0.109		0.622	0.046	0.475	0.729	0.202	0.014	0.066	0.342
rs174616	0.287	0.001	0.204	0.035	0.016	0.031	0.227	0.014	0.123	0.121	0.622		0.068	0.323	0.68	0.125	0.043	0.035	0.369
rs482548	0.047	0.029	0.02	0.014	0	0.008	0.029	0.003	0.021	0.033	0.046	0.068		0.024	0.053	0.009	0.004	0.005	0.032
rs174627	0.232	0	0.486	0.013	0.008	0.006	0.371	0	0.005	0.033	0.475	0.323	0.024		0.408	0.029	0.017	0.001	0.484
rs174448	0.363	0.004	0.261	0.037	0.015	0.037	0.312	0.022	0.199	0.049	0.729	0.68	0.053	0.408		0.174	0.098	0.053	0.568
rs7482316	0.067	0.003	0.009	0.124	0.154	0.191	0.001	0.031	0.094	0.025	0.202	0.125	0.009	0.029	0.174		0	0.283	0.02
rs7942717	0.021	0.002	0.002	0.008	0.007	0.018	0.004	0.011	0.085	0.002	0.014	0.043	0.004	0.017	0.098	0		0.003	0.161
rs7394871	0.037	0.002	0.012	0.177	0.357	0.249	0.018	0.002	0.013	0.017	0.066	0.035	0.005	0.001	0.053	0.283	0.003		0.02
rs174456	0.222	0.001	0.292	0.001	0.002	0	0.292	0.002	0.034	0.022	0.342	0.369	0.032	0.484	0.568	0.02	0.161	0.02

**Table 4:** Linkage disequilibrium (*R* ^2^) of the tagging SNPs within the *ELOVL2* gene

	rs4532436	rs12195587	rs2281591	rs911196	rs976081	rs13204015
rs4532436		0.169	0.372	0.301	0.237	0.061
rs12195587	0.169		0.063	0.051	0.056	0.297
rs2281591	0.372	0.063		0.08	0.088	0.023
rs911196	0.301	0.051	0.08		0.071	0.019
rs976081	0.237	0.056	0.088	0.071		0.001
rs13204015	0.061	0.297	0.023	0.019	0.001

**Table 5:** Linkage disequilibrium (*R* ^2^) of the tagging SNPs within the *ELOVL5* gene cluster

	rs2073040	rs2294852	rs9370194	rs209492
rs2073040		0.576	0.737	0.153
rs2294852	0.576		0.423	0.067
rs9370194	0.737	0.423		0.02
rs209492	0.153	0.067	0.02

**Table 6:** δ-5 Desaturase indexes (AA:DGLA) after a 6-week n-3 PUFA supplementation according to genotype for each SNPs of the *FADS* gene cluster (n = 208)

	Pre-n-3 PUFA D5D activity^a^			Post-n-3 PUFA D5D activity^a^			*p* values		
	11	12 or 12 + 22	22	11	12 or 12 + 22	22	SNPs	Suppl.	Interaction
*FADS1*									
rs174546	4.132 ± 1.179	3.166 ± 0.807	2.376 ± 0.572	4.787 ± 1.138	4.091 ± 1.123	3.086 ± 0.739	<0.0001	<0.0001	0.20
*FADS2*									
rs498793	3.457 ± 1.413	3.567 ± 1.076	3.816 ± 0.815	4.317 ± 1.291	4.383 ± 1.178	4.304 ± 1.391	0.09	<0.0001	0.63
rs174579	3.860 ± 1.222	3.130 ± 0.886	–	4.575 ± 1.282	3.981 ± 1.108	–	<0.0001	<0.0001	0.32
rs174611	2.934 ± 0.760	3.209 ± 0.949	3.919 ± 1.226	4.544 ± 1.152	4.167 ± 1.385	3.735 ± 0.666	<0.0001	<0.0001	0.08
rs174616	3.272 ± 1.203	3.563 ± 1.162	3.904 ± 0.019	4.485 ± 1.098	4.469 ± 1.298	3.948 ± 1.129	0.04	<0.0001	0.09
*FADS3*									
rs174456	3.706 ± 0.993	3.377 ± 1.106	3.767 ± 1.951	4.468 ± 1.258	4.187 ± 1.154	4.436 ± 1.592	0.07	<0.0001	0.86
Intergenic regions									
rs174627	3.732 ± 1.200	3.080 ± 0.847	–	4.444 ± 1.251	4.017 ± 1.194	–	0.0006	<0.0001	0.14
rs12807005	3.569 ± 1.150	3.700 ± 1.547	–	4.360 ± 1.257	3.609 ± 0.488	–	0.56	0.02	0.07
rs174448	3.859 ± 0.990	3.526 ± 1.268	2.800 ± 0.717	4.539 ± 1.113	4.327 ± 1.372	3.681 ± 0.792	0.001	<0.0001	0.59

Repeated MIXED procedure implemented in S.A.S statistical software v. 9.3 adjusted for age, sex and BMI

^a^Values are mean ± SD

**Table 7:** δ-6 Desaturase indexes (DGLA:LA) after a 6-week n-3 PUFA supplementation according to genotype for each SNPs of the *FADS* gene cluster

	Pre-n-3 PUFA D6D activity^a^			Post-n-3 PUFA D6D activity^a^			*p* values		
	11	12 or 12 + 22	22	11	12 or 12 + 22	22	SNPs	Suppl.	Interaction
*FADS1*									
rs174546	0.160 ± 0.043	0.179 ± 0.046	0.180 ± 0.054	0.132 ± 0.038	0.143 ± 0.042	0.142 ± 0.041	0.02	<0.0001	0.23
*FADS2*									
rs498793	0.172 ± 0.049	0.169 ± 0.042	0.169 ± 0.054	0.138 ± 0.044	0.134 ± 0.037	0.145 ± 0.045	0.64	<0.0001	0.51
rs174579	0.166 ± 0.044	0.176 ± 0.048	–	0.135 ± 0.041	0.141 ± 0.040	–	0.04	<0.0001	0.23
rs174611	0.165 ± 0.050	0.179 ± 0.048	0.163 ± 0.043	0.137 ± 0.039	0.139 ± 0.043	0.132 ± 0.035	0.23	<0.0001	0.01
rs174616	0.178 ± 0.045	0.168 ± 0.048	0.164 ± 0.043	0.139 ± 0.036	0.132 ± 0.041	0.147 ± 0.043	0.34	<0.0001	0.11
*FADS3*									
rs174456	0.166 ± 0.042	0.172 ± 0.051	0.177 ± 0.044	0.136 ± 0.039	0.137 ± 0.041	0.143 ± 0.048	0.73	<0.0001	0.57
Intergenic regions									
rs174627	0.167 ± 0.045	0.178 ± 0.048	–	0.137 ± 0.041	0.137 ± 0.037	–	0.29	<0.0001	0.02
rs12807005	0.170 ± 0.046	0.177 ± 0.035	–	0.137 ± 0.041	0.163 ± 0.033	–	0.29	<0.0001	0.20
rs174448	0.162 ± 0.041	0.172 ± 0.049	0.184 ± 0.047	0.135 ± 0.037	0.139 ± 0.044	0.140 ± 0.037	0.30	<0.0001	0.09

Repeated MIXED procedure implemented in S.A.S statistical software v. 9.3 adjusted for age, sex and BMI

^a^Values are mean ± SD

**Table 8:** Index of elongase activity (22:4n-6/20:4n-6) after a 6-week n-3 PUFA supplementation according to genotype for each SNPs of the *ELOVL* gene family

	Pre-n-3 PUFA index of elongase activity^a^			Post-n-3 PUFA index of elongase activity^a^			*p* values		
	11	12 or 12 + 22	22	11	12 or 12 + 22	22	SNPs	Suppl.	Interaction
*ELOVL2*									
rs13204015	3.06E−2±0.53E−2	3.20E−2±0.65E−2	–	2.09E−2±0.64E−2	1.92E−2±0.96E−2	–	0.89	<0.0001	0.15
rs12195587	3.23E−2±0.62E−2	3.08E−2±0.69E−2	–	1.88E−2±0.96E−2	2.04E−2±0.86E−2	–	0.98	0.0005	0.02
rs4532436	3.01E−2±0.49E−2	3.18E−2−2±0.66E−2	3.40E−2±0.68E−2	1.89E−2±0.75E−2	1.97E−2±0.96E−2	1.86E−2±1.04E−2	0.52	<0.0001	0.05
rs3734397	3.20E−2±0.67E−2	3.22E−2±0.56E−2	2.87E−2±0.65E−2	1.93E−2±0.99E−2	2.00E−2±0.81E−2	1.51E−2±0.95E−2	0.11	<0.0001	0.85
rs2281591	3.20E−2±0.62E−2	3.11E−2±0.65E−2	3.64E−2±0.52E−2	2.05E−2±0.89E−2	1.76E−2±0.95E−2	2.22E−2±0.99E−2	0.01	<0.0001	0.22
rs3798710	3.14E−2±0.61E−2	3.29E−2±0.69E−2	–	1.89E−2±0.90E−2	2.01E−2±1.00E−2	–	0.06	<0.0001	0.83
*ELOVL5*									
rs209492	3.17E−2−2±0.62E−2	3.24E−2±0.69E−2	–	1.91E−2±0.92E−2	1.99E−2±0.98E−2	–	0.45	<0.0001	0.94
rs2073040	3.18E−2±0.65E−2	3.19E−2±0.61E−2	3.22E−2±0.72E−2	1.83E−2±1.05E−2	1.97E−2±0.87E−2	2.07E−2±0.75E−2	0.63	<0.0001	0.43
rs2294852	3.15E−2±0.73E−2	3.22E−2±0.60E−2	3.16E−2±0.61E−2	1.84E−2±1.01E−2	1.95E−2±0.96E−2	2.00E−2±0.78E−2	0.83	<0.0001	0.62
rs9370194	3.21E−2±0.67E−2	3.17E−2±0.61E−2	–	1.87E−2±1.07E−2	1.98E−2±0.79E−2	–	0.98	<0.0001	0.20

Repeated MIXED procedure implemented in S.A.S statistical software v. 9.3 adjusted for age, sex, and BMI

^a^Values are mean ± SD

**Fig. 2:**
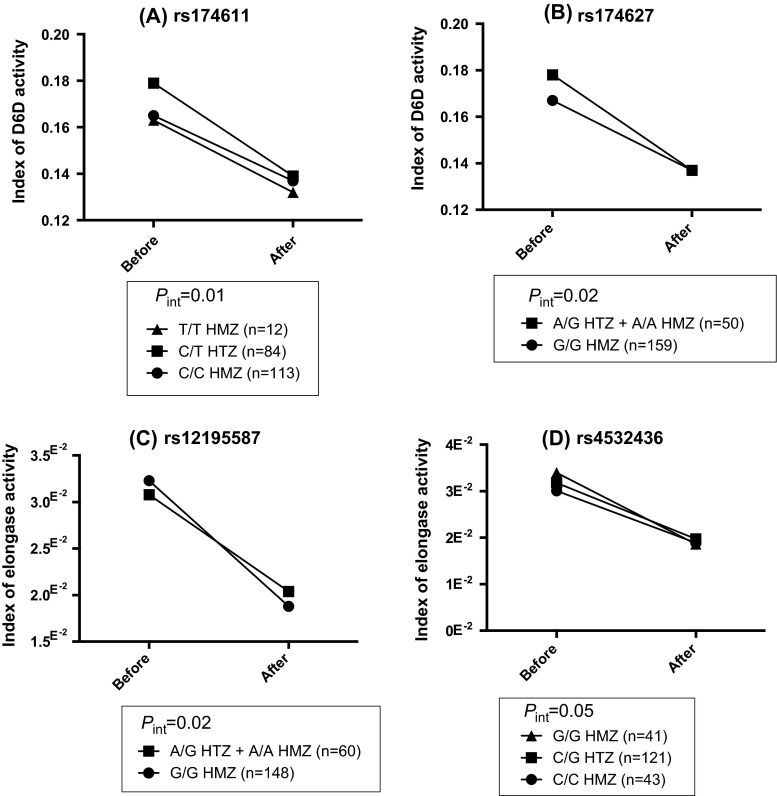
Gene–diet interaction effects modulating indexes of enzyme activities after an n-3 fatty acid supplementation

In a MIXED model for repeated measures including the effects of time (supplementation), genotype and the interaction (genotype × supplementation) adjusted for age, sex and BMI, ten SNPs of the *FADS* gene cluster (rs174546, rs2072114, rs2845573, rs174570, rs174579, rs174611, rs174616, rs968567, rs174627 and rs174448) were associated with estimates of D5D activity (*p* < 0.04, for all). Three SNPs were associated with surrogate estimates of D6D activity (rs174546, rs175579 and rs968567, *p* < 0.04, for all), and one SNP was associated with the index of elongase activity: rs2281591 (*p* = 0.01) (Tables 6, 7, 8). Results in Tables 6, 7, and 8 were only provided for frequent SNPs with MAFs >20 % and/or for significant gene–diet interaction effects. Some gene–diet interaction effects with SNPs within the *FADS* gene cluster or the *ELOVL* gene family were found and could potentially modulate the enzyme activities of desaturases and elongases following the n-3 FA supplementation. Two gene–diet interactions on D6D activities were found with SNPs of the *FADS* gene cluster for rs174611 (*p* = 0.01) and rs174627 (*p* = 0.02) after the n-3 FA supplementation, and two gene–diet interactions on the elongase activity were also found with SNPs of the *ELOVL* gene family for rs12195587 (*p* = 0.02) and rs4532436 (*p* = 0.05) (Fig. 2).

## Discussion

In this study, we tested whether a 6-week supplementation with high doses of n-3 FA above 3 g/day led to differences in enzyme activities expressed as surrogate estimates of D5D and D6D activities and index of elongase activity, as well as if SNPs of genes encoding for these enzymes modulate the enzyme activity following a 6-week n-3 FA supplementation. To do so, 28 SNPs were selected from five genes (*FADS1, FADS2, FADS3, ELOVL2* and *ELOVL5*) covering at least 85 % of the common genetic variations.

In the present study, a decrease in plasma levels of n-6 FAs (LA, DGLA, AA and adrenic acid) was observed as well as a decrease in plasma levels of ALA, an essential n-3 FA that is also the precursor for the synthesis of EPA and DHA. Subsequently, an increase in the estimate of D5D activity and a decrease in the estimate of D6D activity and the index of elongase activity were observed. D5D activity was negatively associated with cardio-metabolic risk factors, while D6D activity was positively associated. Minor alleles of some SNPs in the *FADS* gene cluster are associated with higher D6D activity and with lower D5D activity in French Canadians while minor allele of SNPs from the *ELOVL* gene family are associated with higher elongase activity following a 6-week supplementation with 5 g/day of fish oil.

The endogenous synthesis of LC-PUFAs is made possible by concerted actions between elongases and desaturases. Index of elongase activity has been much less extensively studied in the context of CVD than desaturase activity was, but Sethom et al. (2011) have shown that elongase and D5D activities tended to decrease according to the number of metabolic syndrome features in Tunisian patients (Sethom et al. 2011). In the current study, we observed a significant increase of D5D activity following an n-3 FA supplementation meaning that the n-3 FA supplementation may exert beneficial effects on CVD risk factors thus potentially impacting on obesity and metabolic syndrome features. A recent study suggested that fatty acid elongase-5 (encoded by *ELOVL5* gene) plays a key role in regulating hepatic lipid and carbohydrate metabolism (Wang et al. 2008). Fatty acid elongase-5 elongates γ-linolenic acid (GLA, C18:3n-6) to form DGLA (C20:3n-6) which is converted to AA (C20:4n-6) by the D5D (*FADS1*) in the n-6 metabolism (Moon et al. 2009; Qin et al. 2009).

There is a competition between ALA and C24:5n-3 (tetracosapentaenoic acid) for the D6D, which could lead to decreased levels of DHA after certain intakes of dietary ALA (Gregory et al. 2011). However, in the present study, EPA + DHA was given directly via fish oil supplements, increasing considerably DHA intake and favoring the incorporation of n-3 FA in plasma phospholipids at the expense of the incorporation of n-6 s. Thus, we hypothesized that the D6D (*FADS2*) is more likely to be used to convert ALA in C18:4n-3 as shown by decreased ALA levels in plasma phospholipids (Table 1). Gregory et al. (2011)also observed a saturation of the second FA elongase-2 reaction (DPA, C22:5n-3 → C24:5n3) that could potentially explain the accumulation of DPA when EPA is provided in the diet. In the present study, plasma phospholipid EPA levels increased by 330.0 ± 214.1 % after the 6-week supplementation and DPA increased by 49.0 ± 36.8 % (Table 1).

Since D6D is the rate-limiting enzyme in the PUFA metabolism (Sprecher 2002) and is not specific to a single pathway, one could pretend that giving high doses of n-3 possibly activates the n-3 FA pathway and favors the incorporation of n-3 FAs at the expense of the incorporation of n-6 FAs. Moreover, the use of D6D twice in the conversion of LC-PUFAs suggests that this enzyme may play a key regulatory role in the PUFA metabolism (Portolesi et al. 2008).

In the present study, we found two gene–diet interactions with SNPs from the *FADS* gene cluster and the n-3 FA supplementation modulating surrogate estimates of D6D activity where carriers of the minor allele had the lowest activity. Warensjo et al. confirmed that estimates of D5D activity are inversely related to obesity and insulin resistance, whereas D6D activity shows positive associations (Warensjo et al. 2009). Increased estimates of D6D activities and decreased estimates of D5D and elongase activities were also associated with adverse profiles of several metabolic risk factors in a group of free-living young Japanese women (Murakami et al. 2008). After the 6-week n-3 FA supplementation, we observed an increase in the estimates of D5D activity and a decrease in the estimates of D6D activity meaning that n-3 FA supplements may be beneficial in the prevention of obesity and insulin resistance. SNPs within the *FADS* gene cluster are believed to alter desaturase activity as shown in a recent paper by Merino et al. (2011), especially rs175547 (in high LD with rs174546, *r* ^2^ = 1.0) thought to be a causal SNP in the *FADS* gene cluster (Merino et al. 2011). Herein, minor allele homozygotes of all SNPs (or minor allele homozygotes + heterozygotes for SNPs included in the dominant model) that were associated with estimates of D5D activity always exhibited the lowest desaturase activity after the 6-week n-3 FA supplementation, suggesting that they may be at higher risk to develop obesity-related metabolic complications.

Previous studies have also reported associations between SNPs within the *FADS* gene cluster and estimates of desaturase activities (Bokor et al. 2010; Martinelli et al. 2008). Recently, Gillingham et al. (2013) have studied the effects of diets enriched in flaxseed oil or high-oleic acid canola oil and SNPs from *FADS1*-*2* genes on plasma FAs and found several SNPs associated with [U-^13^C]ALA metabolism, a precise measure of desaturase activity. Al-Hilal et al. (2013) have shown that genotypes of three SNPs located in the *FADS1/FADS2* gene cluster were strongly associated with proportions of LC-PUFAs and desaturase activities estimated in plasma and in erythrocytes. The same research group has also shown that a higher EPA + DHA dosage reduced n-6 and increased n-3 LC-PUFA proportions and that D5D desaturase activity increased which is concomitant with results observed here (Al-Hilal et al. 2013). In the present study, we have found three significant gene–diet interactions after a 6-week n-3 FA supplementation. In addition, some SNPs were associated with indexes of desaturase or elongase activities independently of the supplementation.

### Strengths and limitations

Indexes of desaturase and elongase activities were estimated using product-to-precursor ratios as surrogate markers of desaturase or elongase activities, because direct measures were not possible. The quantification of the GLA FA was not feasible resulting from low proportions of that specific FA in plasma phospholipids. Although there is an elongation step comprise in the (20:3n-6)/(18:2n-6) ratio, it should be noted that this is not considered as the rate-limiting step in the n-6 Fas synthesis. Consequently, the (20:3n-6)/(18:2n-6) ratio was considered as good estimates and was used to assess the estimate of D6D activity (Bokor et al. 2010; Krachler et al. 2008; Warensjo et al. 2008). Indexes of desaturase and elongase activities depend not only on enzyme activities, but also on PUFA intake.

## Conclusions

In summary, a 6-week fish oil supplementation decreased plasma levels of n-6 and increased plasma n-3 FA levels impacting desaturase and elongase activities. Genetic predispositions on genes coding for these enzymes may lead to more or less LC-PUFA conversion depending on the genotype. Some SNPs of the *FADS* gene cluster and the *ELOVL* gene family may play an important role in the enzyme activity of desaturases and elongases, suggesting that a supplementation with n-3 FA may affect PUFA metabolism.

## Abbreviations

*FADS1*: Fatty acid desaturase 1
*FADS2*: Fatty acid desaturase 2
*FADS3*: Fatty acid desaturase 3
*ELOVL2*: Fatty acid elongase 2
*ELOVL5*: Fatty acid elongase 5
n-3: Omega-3
n-6: Omega-6
n-9: Omega-9
MAF: Minor allele frequency
ALA: Alpha-linolenic
D5D: δ-5 Desaturase
D6D: δ-6 Desaturase
DHA: Docosahexaenoic acid
EPA: Eicosapentaenoic acid
AA: Arachidonic acid
LC: Long chain
UTR: Untranslated region
FA: Fatty acid
PUFA: Polyunsaturated fatty acids
BMI: Body mass index
DGLA: Dihomo-γ-linolenic acid
LA: Linoleic acid
GLA: γ-Linolenic acid

## References

[CR1] Al-Hilal M (2013). Genetic variation at the FADS1-FADS2 gene locus influences delta-5 desaturase activity and LC-PUFA proportions after fish oil supplement. J Lipid Res.

[CR2] Bokor S (2010). Single nucleotide polymorphisms in the FADS gene cluster are associated with delta-5 and delta-6 desaturase activities estimated by serum fatty acid ratios. J Lipid Res.

[CR3] Calder PC (2009). Polyunsaturated fatty acids and inflammatory processes: new twists in an old tale. Biochimie.

[CR4] Cormier H (2012). Association between polymorphisms in the fatty acid desaturase gene cluster and the plasma triacylglycerol response to an n-3 PUFA supplementation. Nutrients.

[CR5] D’Andrea S (2002). The same rat Delta6-desaturase not only acts on 18-but also on 24-carbon fatty acids in very-long-chain polyunsaturated fatty acid biosynthesis. Biochem J.

[CR6] Das UN (2007). A defect in the activity of Delta6 and Delta5 desaturases may be a factor in the initiation and progression of atherosclerosis. Prostaglandins Leukot Essent Fatty Acids.

[CR7] Eating Well with Canada’s Food Guide (2007) Health Canada 10.1111/j.1753-4887.2007.tb00295.x 17503710

[CR9] Gillingham LG, Harding SV, Rideout TC, Yurkova N, Cunnane SC, Eck PK, Jones PJ (2013). Dietary oils and FADS1-FADS2 genetic variants modulate [13C]alpha-linolenic acid metabolism and plasma fatty acid composition. Am J Clin Nutr.

[CR10] Gregory MK, Gibson RA, Cook-Johnson RJ, Cleland LG, James MJ (2011). Elongase reactions as control points in long-chain polyunsaturated fatty acid synthesis. PLoS ONE.

[CR11] Harris WS (2006). The omega-6/omega-3 ratio and cardiovascular disease risk: uses and abuses. Current atherosclerosis reports.

[CR12] Jakobsson A, Westerberg R, Jacobsson A (2006). Fatty acid elongases in mammals: their regulation and roles in metabolism. Prog Lipid Res.

[CR13] Krachler B (2008). Fatty acid profile of the erythrocyte membrane preceding development of type 2 diabetes mellitus. Nutr Metabol Cardiovasc Dis NMCD.

[CR14] Lemaitre RN (2011). Genetic loci associated with plasma phospholipid n-3 fatty acids: a meta-analysis of genome-wide association studies from the CHARGE Consortium. PLoS Genet.

[CR15] Lepage G, Roy CC (1986). Direct transesterification of all classes of lipids in a one-step reaction. J Lipid Res.

[CR16] Livak KJ (1999). Allelic discrimination using fluorogenic probes and the 5′ nuclease assay. Genet Anal Biomol Eng.

[CR17] Malerba G (2008). SNPs of the FADS gene cluster are associated with polyunsaturated fatty acids in a cohort of patients with cardiovascular disease. Lipids.

[CR18] Martinelli N (2008). FADS genotypes and desaturase activity estimated by the ratio of arachidonic acid to linoleic acid are associated with inflammation and coronary artery disease. Am J Clin Nutr.

[CR19] Martinelli N, Consoli L, Olivieri O (2009). A ‘desaturase hypothesis’ for atherosclerosis: Janus-faced enzymes in omega-6 and omega-3 polyunsaturated fatty acid metabolism. J Nutrigenet Nutrigenomics.

[CR20] Merino DM (2011). Polymorphisms in FADS1 and FADS2 alter desaturase activity in young Caucasian and Asian adults. Mol Genet Metab.

[CR21] Moon YA, Hammer RE, Horton JD (2009). Deletion of ELOVL5 leads to fatty liver through activation of SREBP-1c in mice. J Lipid Res.

[CR22] Murakami K (2008). Lower estimates of delta-5 desaturase and elongase activity are related to adverse profiles for several metabolic risk factors in young Japanese women. Nutr Res.

[CR23] Portolesi R, Powell BC, Gibson RA (2007). Competition between 24:5n-3 and ALA for Delta 6 desaturase may limit the accumulation of DHA in HepG2 cell membranes. J Lipid Res.

[CR24] Portolesi R, Powell BC, Gibson RA (2008). Delta6 desaturase mRNA abundance in HepG2 cells is suppressed by unsaturated fatty acids. Lipids.

[CR25] Qin Y, Dalen KT, Gustafsson JA, Nebb HI (2009). Regulation of hepatic fatty acid elongase 5 by LXRalpha-SREBP-1c. Biochim Biophys Acta.

[CR26] Sethom MM (2011). Plasma fatty acids profile and estimated elongase and desaturases activities in Tunisian patients with the metabolic syndrome. Prostaglandins Leukot Essent Fatty Acids.

[CR27] Shaikh NA, Downar E (1981). Time course of changes in porcine myocardial phospholipid levels during ischemia. A reassessment of the lysolipid hypothesis. Circ Res.

[CR28] Simopoulos AP (2008). The importance of the omega-6/omega-3 fatty acid ratio in cardiovascular disease and other chronic diseases. Exp Biol Med.

[CR29] Sprecher H (2002). The roles of anabolic and catabolic reactions in the synthesis and recycling of polyunsaturated fatty acids. Prostaglandins Leukot Essent Fatty Acids.

[CR30] Tanaka T (2009). Genome-wide association study of plasma polyunsaturated fatty acids in the InCHIANTI Study. PLoS Genet.

[CR31] Wang Y, Torres-Gonzalez M, Tripathy S, Botolin D, Christian B, Jump DB (2008). Elevated hepatic fatty acid elongase-5 activity affects multiple pathways controlling hepatic lipid and carbohydrate composition. J Lipid Res.

[CR32] Warensjo E, Riserus U, Gustafsson IB, Mohsen R, Cederholm T, Vessby B (2008). Effects of saturated and unsaturated fatty acids on estimated desaturase activities during a controlled dietary intervention. Nutr Metab Cardiovasc Dis NMCD.

[CR33] Warensjo E, Rosell M, Hellenius ML, Vessby B, De Faire U, Riserus U (2009). Associations between estimated fatty acid desaturase activities in serum lipids and adipose tissue in humans: links to obesity and insulin resistance. Lipids Health Dis.

